# Metabolic shift in the emergence of hyperinvasive pandemic meningococcal lineages

**DOI:** 10.1038/srep41126

**Published:** 2017-01-23

**Authors:** Eleanor R. Watkins, Martin C. J. Maiden

**Affiliations:** 1 Department of Zoology, University of Oxford, South Parks Road, Oxford, OX1 3PS, UK

## Abstract

Hyperinvasive lineages of *Neisseria meningitidis*, which persist despite extensive horizontal genetic exchange, are a major cause of meningitis and septicaemia worldwide. Over the past 50 years one such lineage of meningococci, known as serogroup A, clonal complex 5 (A:cc5), has caused three successive pandemics, including epidemics in sub-Saharan Africa. Although the principal antigens that invoke effective immunity have remained unchanged, distinct A:cc5 epidemic clones have nevertheless emerged. An analysis of whole genome sequence diversity among 153 A:cc5 isolates identified eleven genetic introgression events in the emergence of the epidemic clones, which primarily involved variants of core genes encoding metabolic processes. The acquired DNA was identical to that found over many years in other, unrelated, hyperinvasive meningococci, suggesting that the epidemic clones emerged by acquisition of pre-existing metabolic gene variants, rather than ‘virulence’ associated or antigen-encoding genes. This is consistent with mathematical models which predict the association of transmission fitness with the emergence and maintenance of virulence in recombining commensal organisms.

Although *N. meningitidis* gives rise to 1.2 million cases of meningitis and severe sepsis disease each year^1^, asymptomatic colonisation of the human oropharynx is common, with population carriage rates of 10–30%^2^. As invasive disease does not contribute to person-to-person transmission, the meningococcus is an example of an ‘accidental’ pathogen^3^. Carried meningococci are highly diverse at loci encoding both antigens and metabolic functions, with much of this diversity generated by genetic reassortment, mediated by horizontal genetic transfer (HGT). Despite this diversity, meningococcal populations are highly structured into distinct genealogical groups or lineages, which are recognised by multilocus sequence typing (MLST) as ‘clonal complexes’ (ccs), which comprise closely related sequence types (STs)^4^. Epidemic meningococcal disease is caused by a subset of ccs, the ‘hyperinvasive lineages’, which persist for decades and during geographical spread^5^,^6^. Notwithstanding their propensity to cause invasive disease, hyperinvasive meningococci must also be efficient at asymptomatic transmission and several theoretical frameworks have been proposed to explain their emergence and persistence, including ‘strain structure theory’ which posits that the invasive phenotype can be stable in highly transmissible lineages^3^.

Since 1905, many of the largest recorded epidemics of meningococcal disease have occurred in the sub-Saharan African Meningitis Belt, mostly caused by serogroup A meningococci belonging to cc1, cc4, and cc5 (A:cc1; A:cc4; and A:cc5; Figure S1)^7^. These meningococci have also been responsible for a number of pandemics throughout the 20^th^ century^8^, with A:cc1 meningococci dominant in Africa until they were replaced by A:cc5 meningococci during the Hajj-associated epidemics of 1987, part of the third A:cc5 global pandemic. Since that time, A:cc5 organisms have caused successive epidemics in the meningitis belt^8^ (Fig. 1A), including a large outbreak in 1997–1998, with more than 250,000 cases^9^. A:cc5 epidemic across the belt in the early 2000s coincided with the emergence of the novel cc5 genotypes A:cc5:ST-7 and A:cc5:ST-2859, which were identical to the original genotype (A:cc5:ST-5) at their principal antigens^10^,^11^,^12^. These disease outbreaks in the African Meningitis Belt prompted the development and implementation of the serogroup A conjugate vaccine PsA-TT (MenAfriVac^TM^)^13^, a polysaccharide–tetanus toxoid conjugate vaccine which targets the polysaccharide capsule of serogroup A meningococci.

The reasons for repeated pandemics and epidemics of antigenically highly uniform but distinct serogroup A meningococci remain poorly understood. Such marked levels of genetic and antigenic uniformity are unusual among meningococci, which exhibit high levels of genetic and antigenic diversity^14^ and the global spread of A:cc5 meningococcal variants provides an opportunity to study the emergence of new pandemic lineages with limited genetic variation, in an organism which is usually highly diverse as a consequence of extensive HGT. Understanding this stability has important implications for the continued use of the PsA-TT vaccine, the success of which depends on the antigenic stability of hyperinvasive serogroup A meningococci^13^. Using a gene-by-gene whole genome MLST (wgMLST) approach, we analysed 153 sets of WGS data from A:cc5 meningococci representative of the various A:cc5 pandemics and epidemics, to elucidate the diversification of this lineage over time and to investigate possible genetic factors driving the evolution of distinct variants.

## Results and Discussion

**Figure 1:**
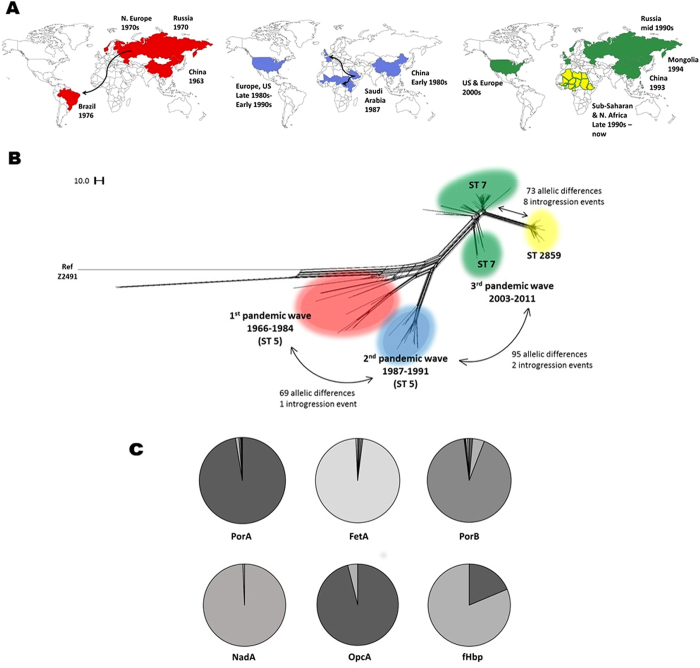
Geographic, genetic and antigenic diversity of 153 serogroup A isolates belonging to the ST-5 complex. (**A**) Global spread of the ST-5 complex in three successive pandemics (red: first pandemic wave; blue: second pandemic wave; green: third pandemic wave; yellow: ST-2859). (**B**) Allele-based phylogenetic network of 153 whole genomes of the ST-5 complex. (**C**) Pie-charts of allelic diversity of six major sub-capsular antigens across the 153 isolates. Most isolates had a highly consistent antigenic profile, with a single dominant allele found for each antigen (PorA: 97.4% = allele P1.20,9; FetA: 97.4% = allele F3-1; PorB: 92.1% = allele 3–47; NadA: 99.3% = allele 7; OpcA: 96.1% = allele 3; fHbp: 83.1% = allele 39). PorA, FetA, PorB, NadA and fHbp have all been shown to induce an immune response and deployed in various protein-based vaccines^15^,^16^. See also Table S7. The maps were created using mapchart.net (www.mapchart.net).

We refer to the three A:cc5 pandemics and the outbreaks caused by A:cc5:ST-2859 as four separate “epidemic waves”. There was allelic variation at 50.1% (999/1993) of loci across the 153 meningococcal genomes analysed, but within epidemic waves the genomes were highly uniform, with an average of 67.2% of loci exhibiting identical alleles within each wave, and 87.2% of variants exhibiting alleles which were identical across >90% of all alleles within each wave. Eight major immunogenic sub-capsular antigens, many of which have been used in licensed protein-based meningococcal vaccines^15^,^16^, were highly conserved, with >99% of the A:cc5 meningococci exhibiting identical alleles (Fig. 1C; Table S7). Previous studies identified differences in six antigens (transferrin-binding protein B, IgA1 protease, OpaB, OpaD, FetA) and the *lgt* gene (involved in lipooligopolysaccharide synthesis) between isolates of the second and third pandemics^8^,^17^,^18^,^19^, and differences in Maf adhesins and pilin glycosylation loci among ST-7 and ST-2859 isolates^20^. The fact that the A:cc5 meningococci isolates manifest identical alleles at loci encoding six major antigens, which are known to elicit protective immune responses as assessed by serum bactericidal antibodies^21^, and which are also known to vary among meningococci to avoid protective immunity in Africa and elsewhere^22^, suggests that the evasion of host immune responses is unlikely to have played a major role in the emergence of the epidemic waves in Africa.

**Figure 2:**
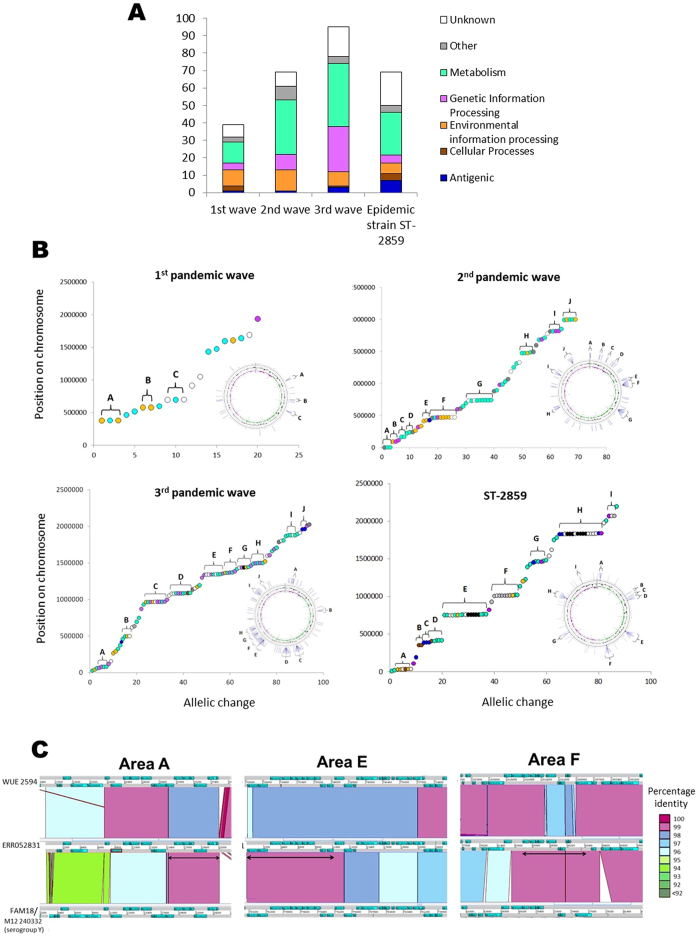
Functional characterisation and location of alleles specific to each epidemic wave. (**A**) Functional characterisations of alleles specific to each epidemic wave. (**B**) Plots of successive allelic changes against their position in the reference genome for each epidemic wave, with an accompanying plot of these changes annotated on the circular chromosome of Z2491 (or WUE2594 for ST-2859). Letters indicate areas of allelic changes which are adjacent on the chromosome. (**C**) Genomic areas of putative recombination between ST-2859 and ST-11/ST-167 strains. Comparison plots show hypothetical donor strains on the bottom level (either reference strain *FAM18*, an isolate of serogroup C ST-11, or *M12 240332*, a serogroup Y ST-167 complex strain), ST-2859 on the central level (isolate *ERR052831*), and reference strain *WUE 2594* (serogroup A, ST-5 complex) on the top level (representative of the recipient strain). The arrows signify putative areas of recombination, and correspond to higher sequence identity shared between *FAM18/M12 240332* and ST-2859, than the ancestral *WUE 2594* strain and ST2859.

Although the genes encoding the major immunogenic antigens were highly conserved, a number of allelic differences were observed among the epidemic waves: there were 39 loci with alleles specific to the first pandemic; 69 loci with alleles specific to the second pandemic; and 95 loci with alleles specific to the third pandemic (Tables S2–4). A total of 73 loci were specific to ST-2859 genomes, with 14 loci absent compared to ST-5 isolates (Fig. 1B; Tables S5–6). The variable loci were annotated as encoding: metabolic functions (40.0%); genetic information processing (16.2%); environmental information processing (12.3%); other functions (6.6%); antigenic genes (3.7%); and cellular processes (2.2%); and there was no characterised function for 18.3% of these loci (Fig. 2A). The variable loci were dispersed around the chromosome, but when plotted consecutively against their position in a reference genome, allelic changes at several contiguous loci were observed (Fig. 2B), suggesting the introgression of large genomic regions (of up to 16.2 kb) from other meningococci via HGT in the emergence of these strains.

These putatively introgressed loci were examined for possible sources of HGT by comparison to allelic variation recorded in the PubMLST.org/neisseria database. Exact nucleotide matches to recorded meningococcal isolates in the database were observed at eight contiguous groups of loci (areas A, C, D, E, F, G, H and I in ST-2859 isolates; areas G and H in second pandemic wave isolates; and area H in third pandemic wave isolates; see Table 1, Fig. 2C). Areas G and H from the second pandemic, as well as Area H in the third pandemic, had exact matches to large numbers of globally distributed isolates belonging to cc11 hyperinvasive meningococci^23^. The oldest matching cc11 isolates dated from 1964, several years in advance of the second A:cc5 pandemic, which was consistent with these introgressions originating in cc11. The regions of contiguous allelic changes within ST-2859 isolates contained exact matches to isolates belonging to multiple hyperinvasive clonal complexes. The alleles from seven of these regions were present in globally-distributed cc11 isolates and were universally present in a group of W:cc11 strains circulating in Burkina Faso and Niger in 2001 and 2002. These W:cc11 isolates therefore represent plausible relatives of the donor strains involved in the emergence of the A:cc5:ST-2859 strain in sub-Saharan Africa. This timescale is consistent with the phylogenetic analyses of Lamelas *et al*., who calculated that the ST-2859 lineage emerged in Burkina Faso in 2000^20^. Area F of A:cc5:ST-2859 also matched isolates from another lineage typically expressing serogroup Y, Y:cc167, with the earliest allele identified in an isolate from 1940. These data are consistent with the epidemic A:cc5:ST-2859 strain arising from multiple introgression events involving several hyperinvasive meningococci in a short time, which accounts for the rapid accumulation of diversity and the long branch lengths in allele-based phylogenies (Fig. 1).

Importantly these introgression events did not involve the major antigens, which are very different in these other lineages, as would be expected in an immune selection model. In addition, most of the introgressed alleles were present in older isolates in the PubMLST database (Table 1), with the earliest dating from 1937, demonstrating that the introgressed alleles are long-lived, having circulated over periods of several decades at least. HGT occurs frequently among carried meningococci^3^,^14^; however, given that the hyperinvasive meningococci represent a small minority of the carried population in Africa^24^, as elsewhere, the acquisition of multiple contiguous identical alleles by one hyperinvasive meningococcus from another, appears to be highly unlikely and is suggestive of a selective process. It is well established that rare HGT events, between and within *Neisseria* species, can be amplified in meningococcal populations by factors such as antibiotic^25^ use and immunological pressure^26^, and the data presented here are consistent with selection for tracts of metabolic gene variants within the A:cc5 genome.

The majority of putatively introgressed genes were annotated as having metabolic functions (62.5%, 25 out of 40 loci; 50% of all 50 loci, including those with unknown functions) (Figure S2). Differences in metabolic genes can contribute to the emergence of epidemic strains in a number of ways. First, there is increasing support for the idea that metabolic genes play important roles in pathogenesis and virulence in both meningococci^27^ and other bacterial pathogens^28^. Such metabolic adaptation could allow meningococci to exploit alternative host resources in invasive disseminated infections^29^, for example, and differences have been shown in the expression of metabolic genes between meningococci adhering to lung epithelial cells and growing in blood^27^,^30^,^31^. It is also plausible that differences in metabolic efficiency lead to differences in transmissibility among strains, as strains assimilate metabolites at different rates. Indeed, a recent study^27^ showed a significantly higher *in vitro* growth rate among meningococcal strains from hyperinvasive lineages compared to those from carried lineages.

Analysis of the predicted metabolic functions of the introgressed genes indicated that the largest category was energy metabolism (30%) (Figure S3). Notably, introgressed area G from isolates of the second pandemic encoded several subunits from the Na^+^-translocating NADH-quinone reductase complex, which carries out key redox reactions of the electron transport pathway, and are predicted to interact in the same functional network (Figure S4). Although we are not aware of any experimental data for the functional significance of Na^+^-translocating NADH-quinone reductase complex in *N. meningitidis*, there are experimental data from other Gram Negative pathogens suggesting that genetic variants of the Na^+^-translocating NADH-quinone reductase complex have different affinities for sodium ions in different species, thus influencing rates of NADH oxidation and energy transduction^32^. It is plausible that differences in energy metabolism among strains from the same species could result in differences in transmission phenotype. Given the importance of rapid growth of meningococci in the blood stream in the development of IMD in individuals, these changes also have implications for virulent phenotypes. Several of the other allelic differences were in loci assigned to genetic information processing functions (16.2%). Further, the functions of the genes in the genetic information processing category pertain to DNA replication, transcription, translation and repair, and it is therefore plausible that allelic differences may influence growth and replication rates^33^,^34^.

A predominant paradigm for changes in the frequency of epidemic clones is that antigenic genes vary over time by diversifying immune selection, while metabolic genes exhibit stabilising selection for conservation of function; indeed, the emergence of virulent strains in pathogenic bacteria has been associated with the import of, or mutation within, antibiotic resistance genes, antigens or virulence factors^20^. An alternate paradigm, however, posits that antigenic genes can be stable over time in non-overlapping combinations, as a consequence of between-host competition^35^. Such non-overlapping patterns have been observed among the PorA, FetA and Opa antigens of *N. meningitidis*, with many identical epitope combinations maintained over several decades^5^,^36^,^37^. By contrast, metabolic genes should evolve over time as a result of intense ecological competition within the host, so as to gain a competitive advantage against strains inhabiting the same antigenic niche: a phenomenon referred to as metabolic shift^38^. Our findings are consistent with mathematical models of immunological and ecological competition in *N. meningitidis* and also *Streptococcus pneumoniae*, which assume that metabolic differences between bacterial strains can lead to small differences in transmission fitness^3^,^38^. Simulations have shown successive replacement over time by strains with increasing metabolic fitness but similar antigenic properties.

Invasive disease is caused by a minority of meningococcal genotypes, the hyperinvasive lineages, which show strong temporal and geographic stability over decades and during global spread. Here, we show that HGT events among diverse long-lived hyperinvasive lineages (*e.g*. introgression into A:cc5:ST-5 from C:cc11:ST-11) can lead to the emergence of new epidemic strains (*i.e*. A:cc5:ST-2859). Our observations suggest that introgression events from one hyperinvasive lineage to another contribute to increased transmission fitness and/or increased virulence leading to the replacement of the previous variant. The introgressed genes, which constitute the majority of genetic differences among epidemic waves, encode metabolic functions, with many of the introgressed alleles present in other hyperinvasive isolates dating back several decades before the emergence of A:cc5:ST-2859.

Although immune escape may have played a minor role, our findings suggest that the most important events in the emergence of the A:cc5:ST-7 and A:cc5:ST-2859 epidemics were the acquisition of metabolic gene variants which have affected complex phenotypes, especially transmission fitness and possibly virulence. Such effects have been postulated in other pathogens^38^ and have potential implications for understanding epidemic bacteria and their prevention by vaccination as they indicate that large scale introgression events that alter the relationship between metabolic and antigenic types occur as a consequence of HGT. In principle such events could be induced by immune selection imposed by mass immunisation against principle antigens and on-going disease surveillance with genomic analysis is required to guard against such an eventuality.

## Methods

The sequence reads for all ST-5 complex meningococcal genomes available as of 17/10/13 were downloaded from the European Nucleotide Archive (http://www.ebi.ac.uk/). The genomes were sequenced at the Wellcome Trust Sanger Institute in Cambridge, UK and the Institute for Genomic Sciences in Maryland, US (Table S1)^20^. Reads were assembled using an automated pipeline based on the Velvet algorithm, version 1.2.01. Annotation was carried out using the “autotagger” feature of the Bacterial Isolates Genome Sequence Database (BIGSdb) software^39^, which scans deposited sequences against defined loci in an automated BLAST process. The whole genome sequence data were compared using the BIGSdb Genome Comparator tool, which is implemented on the PubMLST website (www.pubmlst.org). The coding sequences within the annotation were extracted and compared against the reference strain Z2491 (accession number AL157959) using default parameters. Through Genome Comparator, unique allele sequences were labelled consecutively, allowing the identification of shared and unique alleles between isolates. Loci with alleles specific to each pandemic wave were identified, and functionally characterised according to the KEGG Orthology (KO) groupings of the KEGG database (www.kegg.jp). Genes with uncharacterised functions which did not fall into a KO category were blasted in the PFAM database (www.pfam.sanger.ac.uk), and functionally characterised accordingly. Genes without significant hits in the PFAM search were designated with an “unknown” function. Genome Comparator was run, in addition, with the ST-5 strain WUE 2594 (accession number FR774048) as a reference, to identify alleles specific to the ST-2859 strains.

Allele sequences were blasted against the PubMLST database to find the appropriate NEIS number and allele, which were then scanned in the database for sequence matches in other meningococci. The PubMLST database represents a large repository of whole genome data, with over 3700 whole genomes at the time of writing, from a variety of clonal complexes spanning a 78 year period. The Neighbour Net networks in Figures 1 and S1 were created using SplitsTree v4^40^. Annotated plots of the genome (Fig. 2B) were created using the programme CG view^41^. The Artemis Comparison Tool^42^, funded by the Wellcome Trust, was used to create Fig. 2C.

## Additional Information

**How to cite this article**: Watkins, E. R. and Maiden, M. C. J. Metabolic shift in the emergence of hyperinvasive pandemic meningococcal lineages. *Sci. Rep.* **7**, 41126; doi: 10.1038/srep41126 (2017).

**Publisher's note:** Springer Nature remains neutral with regard to jurisdictional claims in published maps and institutional affiliations.

## Supplementary Material

Supplementary Information

## Figures and Tables

**Table 1:** Loci of putative recombined areas with sequence matches to other hyperinvasive clonal complexes.

	Genome position	Locus in reference genome	Matches on PubMLST database (no. isolates)	Nucleotide differences to match	Clonal complexes of isolates with allelic matches in PubMLST database (no. isolates)	Oldest isolate with allele on PubMLST database	Product	Functional Characterisation
**Isolates belonging to ST**-**2859**								
A	32717	NMAA_0029	513	Exact match	11 (457), 162 (38), 269 (13), 865 (8)	USA, 1964	Thiamine transport system substrate-binding protein	Environmental information processing
A	33812	NMAA_0030	521	Exact match	11 (430), 162 (42), 269 (12), 865 (8), 41/44 (5)	USA, 1964	Mechanosensitive ion channel	Environmental information processing
A	34686	NMAA_0031	926	Exact match	11 (681), 41/44 (43), 162 (41), 269 (27), 35 (18), 167 (15), 60 (9), 461 (9), 282 (8), 865 (8), 213 (6)	USA, 1964	Competence-damaged protein (CinA family)	Unknown
A	35273	NMAA_0032	438	Exact match	11 (418)	USA, 1964	Peptide methionine sulfoxide reductase (putative pilin biogenesis)	Cellular processes
A	36986	NMAA_0033	604	Exact match	11 (544), 269 (32), 865 (6)	USA, 1964	Probable signal recognition particle protein	Environmental information processing
C	389418	NMAA_0322	15	Exact match	11 (15)	UK, 1998	Pilin glycosylation protein pglC	Antigenic
D	409695	NMAA_0337	1046	Exact match	11 (739), 22 (88), 41/44 (56), 167 (20), 8 (19), 18 (16)	The Netherlands, 1963	GTP binding protein engB	Cellular processes
D	410530	NMAA_0338	2585	Exact match	11 (746), 41/44 (521), 269 (374), 23 (262), 32 (135), 22 (88), 162 (47), 35 (41), 174 (36), 167 (31), 8 (21), 18 (18), 198 (12), 282 (11), 60 (10), 213 (5)	Denmark, 1940	Cytochrome C	Metabolism
D	413375	NMAA_0340	839	Exact match	11 (794), 8 (19)	USA, 1964	Cytochrome C biogenesis protein (CcsA)	Metabolism
D	414679	NMAA_0341	1	3 nucleotide differences	11 (1)	UK, 2013	tRNA N6-adenosine threonylcarbamoyltransferase (EC 2.3.1.-)	Metabolism
E	749433	NMAA_0635	153	5 nucleotide differences	41/44 (69), 5 (21), 11 (14), 4 (13)	Burkina Faso, 1963	Ribosomal large subunit pseudouridine synthase F (rluF)	Metabolism
E	750216	NMAA_0636	1068	Exact match	11 (802), 213 (157), 41/44 (20), 254 (10)	Denmark, 1940	ATP-NAD kinase	Metabolism
E	751126	NMAA_0637	1159	Exact match	11 (794), 23 (258), 162 (47), 41/44 (5)	USA, 1964	None	Unknown
E	751743	NMAA_0638	863	Exact match	11 (796), 162 (45)	USA, 1964	NADH(P)-binding	Unknown
E	753236	NMAA_0640	1385	Exact match	11 (812), 41/44 (407), 60 (54), 4 (14), 865 (11), 254 (9), 213 (9)	USA, 1937	UDP-N-acetylenolpyruvoylglucosamine reductase (EC 1.3.1.98)	Metabolism
E	754454	NMAA_0641	842	Exact match	11 (795), 1 (11)	Niger, 1963	Multidrug efflux protein	Environmental information processing
E	756168	NMAA_0642	844	Exact match	11 (602), 41/44 (92)	The Netherlands, 1963	ATP phosphoribosyltransferase regulatory subunit	Metabolism
E	757422	NMAA_0643	362	6 nucleotide differences	11 (820)	USA, 1964	Adenylosuccinate synthetase	Metabolism
E	762625	NMAA_0651	984	Exact match	11 (838), 1 (47), 8 (20), 4 (14), 35 (7), 4821 (5)	USA, 1937	Adenylate kinase	Metabolism
E	764593	NMAA_0653	605	Exact match	11 (586)	USA, 1964	pfkB family carbohydrate kinase (BIGS: D-beta-D-heptose-7-phosphate kinase)	Metabolism
E	765601	NMAA_0654	1343	Exact match	11 (832), 41/44 (434), 8 (20), 22 (5), 269 (5)	USA, 1964	Cytosine-specific methyltransferase	Metabolism
F	1007824	NMAA_0886	10	6 nucleotide differences	167 (7)	UK, 2010	Putative phage tail fiber protein	Other
F	1010855	NMAA_0888	1398	Exact match	41/44 (446), 269 (388), 213 (153), 23 (139), 1, (41), 461 (37), 174 (36), 167 (30), 4 (13)	Burkina Faso, Niger, 1963	Protein of unknown function (DUF497)	Unknown
F	1011222	NMAA_0889	42	Exact match	167 (30). 41/44 (6)	The Netherlands, 1986	Caudovirales tail fibre assembly protein	Other
F	1011806	NMAA_0890	41	Exact match	167 (27), 41/44 (7)	The Netherlands, 1986	None	Unknown
F	1013947	NMAA_0892	1523	Exact match	11 (577), 41/44 (443), 213 (148), 23 (137), 461 (34), 167 (23), 103 (19), 8 (14), 865 (12), 269 (10), 1 (8), 32 (7)	UK, 1941	Putative bacterial lipoprotein (DUF799)	Unknown
F	1014591	NMAA_0893	1091	3 differences	11 (569), 23, (137), 22 (82), 41/44 (43), 1 (42), 53 (35), 35 (30), 167 (30), 8 (8)	Denmark, 1940	None	Unknown
F	1014969	NMAA_0894	146	Exact match	53 (32), 167 (31), 198 (12), 35 (8), 11 (7), 32 (6), 41/44 (5), 269 (5), 1136 (5)	Denmark, 1962	Curli production assembly/transport component CsgG	Environmental information processing
F	1015805	NMAA_0895	203	Exact match	32 (154), 167 (22)	Denmark, 1962	Short chain dehydrogenase	Metabolism
G	1459282	NMAA_1235	796	Exact match	11 (764), 8 (6)	USA, 1964	CTP synthase	Metabolism
G	1461028	NMAA_1236	793	Exact match	11 (768)	USA, 1964	Long-chain-fatty-acid–CoA-ligase	Metabolism
G	1462769	NMAA_1237	691	Exact match	11 (644), 8 (7)	USA, 1964	tRNA (5-methylaminomethyl-2-thiouridylate)-methyltransferase	Genetic information processing
G	1464586	NMAA_1239	2508	Exact match	11 (811), 41/44 (508), 23 (231), 269 (347), 213 (157), 60 (59), 461 (37), 35 (37), 174 (34), 167 (30), 8 (19), 198 (12), 32 (9), 1157 (7), 1136 (5)	Denmark, 1962	Diacylglycerol kinase	Metabolism
H	1829030	NMAA_1551	29	Exact match	11 (28)	Canada, 1970	Adhesin MafB2	Antigenic
H	1832666	NMAA_1557	830	Exact match	11 (807)	Norway, 1969	mafB-CTo1MGI-1 alternative toxic C-terminal extremity	Unknown
H	1837213	NMAA_1566	622	Exact match	11 (601)	USA, 1964	None	Unknown
I	2069801	NMAA_1788	603	Exact match	11 (577), 865 (7)	USA, 1964	Ribosomal RNA small subunit methyltransferase G	Genetic information processing
I	2071324	NMAA_1790	543	Exact match	11 (509), 41/44 (7)	USA, The Netherlands, 1964	Fusaric acid resistance protein family	Metabolism
**Isolates belonging to second pandemic wave**								
G	734253	NMA0741	1288	Exact match	11 (821), 41/44 (313), 22 (85)	USA, 1964	Ubiquinone biosynthesis protein UbiB	Metabolism
G	737825	NMA0745	1053	Exact match	11 (837), 22 (88), 103 (17), 41/44 (10), 5 (5), 4821 (5)	USA, 1964	Putative periplasmic protein	Unknown
G	738066	NMA0746	505	Exact match	11 (346), 22 (84), 32 (5), 4821 (5), 5 (5)	USA, 1964	Thiamine biosynthesis protein	Metabolism
G	739274	NMA0747	959	Exact match	11 (800), 22 (86), 41/44 (5), 32 (5), 4821 (5), 5 (5)	USA, 1964	Na(+)-translocating NADH-quinone reductase subunit F	Metabolism
G	740505	NMA0748	1203	Exact match	11 (805), 22 (76), 41/44 (44), 162 (40), 461 (38), 174 (36), 32 (11), 254 (10), 269 (10), 4821 (5), 5 (5)	USA, 1964	Na(+)-translocating NADH-quinone reductase subunit E	Metabolism
G	741102	NMA0749	1343	Exact match	11 (833), 32 (158), 60 (68), 174 (36), 53 (35), 162 (40), 167 (20), 41/44 (12), 22 (11), 254 (10), 4821 (5), 269 (5)	USA, 1964	Na(+)-translocating NADH-quinone reductase subunit D	Metabolism
G	741728	NMA0750	885	Exact match	11 (798), 41/44 (30), 22 (10)	USA, 1964	Na(+)-translocating NADH-quinone reductase subunit C	Metabolism
H	1476300	NMA1572	46	Exact match	167 (19), 865 (11)	The Netherlands, 1986	Pyridoxamine-5′-phosphate oxidase	Metabolism
H	1477432	NMA1573	42	Exact match	167 (15), 5 (5)	The Netherlands, 1986	Pseudouridine synthase	Metabolism
H	1478271	NMA1574	22	Exact match	167 (14)	The Netherlands, 1986	Integral membrane transporter	Environmental information processing
**Isolates belonging to third pandemic wave**								
H	1496471	NMA1591	634	Exact match	11 (580), 8 (20), 213 (12)	USA, 1964	Type III restriction/modification system enzyme	Genetic Information Processing
H	1499437	NMA1592	765	Exact match	11 (601), 41/44 (83), 8 (19), 5 (10)	Denmark, 1962	L-lactate dehydrogenase	Metabolism
H	1501374	NMA1594	861	Exact match	11 (829), 5 (10)	Denmark, 1962	NifS-like aminotranfserase	Metabolism; Genetic Information Processing

The letters refer to the area of contiguous allelic changes observed on the chromosome.
