# Supplementary material for: Metabolic shift in the emergence of hyperinvasive pandemic meningococcal lineages
Source: Sci Rep. 2017 Jan 23;7:41126. doi: 10.1038/srep41126 (PMC5282872; doi:10.1038/srep41126)
Supplement: Supplementary Information [file srep41126-s1.pdf]

Supplementary Information

Metabolic shift in the emergence of hyperinvasive pandemic meningococcal lineages

Eleanor R. Watkins[1] & Martin C. J. Maiden[1]

[1]Department of Zoology, University of Oxford, South Parks Road, Oxford, OX1 3PS

Supplementary Figures

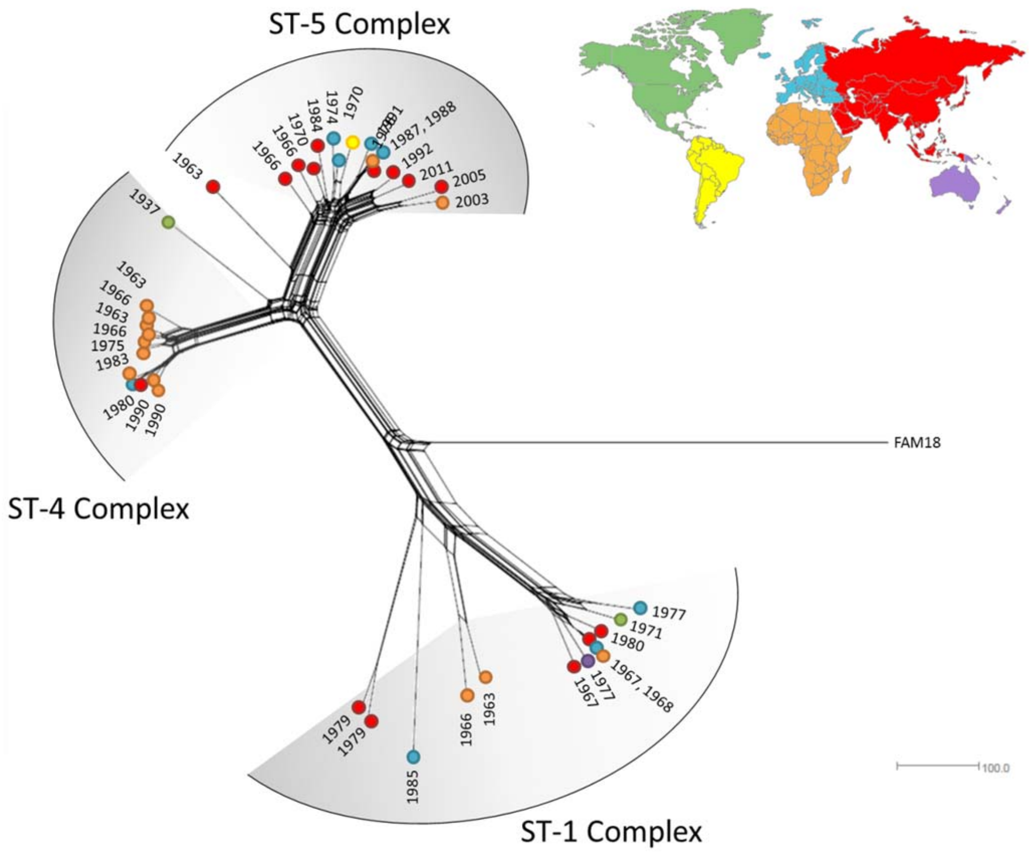

Figure S1. Allele-based phylogenetic network of 41 published serogroup A whole genomes from the ST-1, 4 and 5 complexes. The date of isolation is shown for each strain, and the colour of the tips refers to the continents in which they were isolated. The network was constructed using the NeighbourNet algorithm, based on the alleles of 1993 coding sequences.

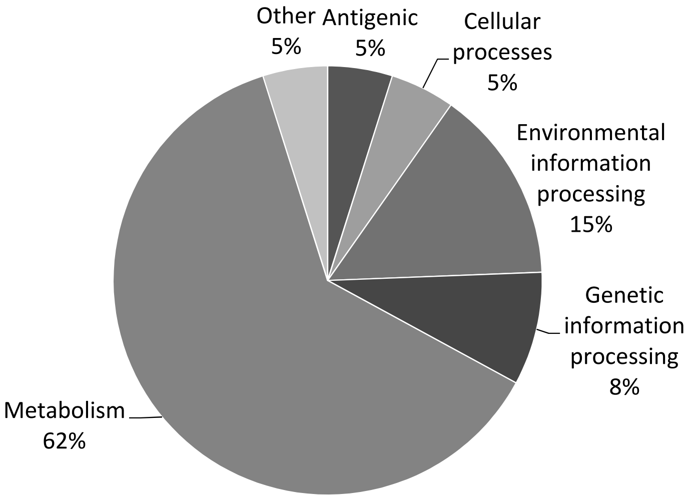

Figure S2. Functional characterisation of the putatively introgressed genes, according to the KEGG database.

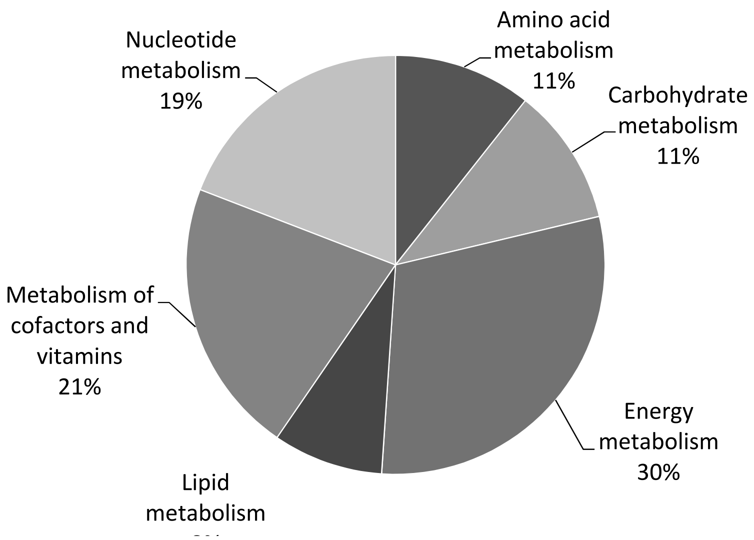

Figure S3. Metabolic functions of the putatively introgressed genes, according to the KEGG database.

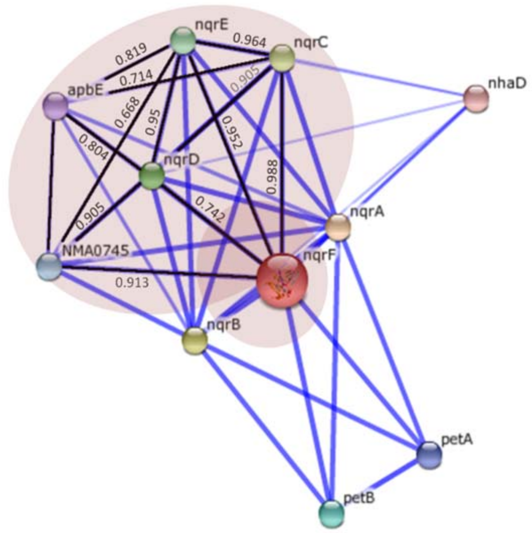

Figure S4. Predicted interactions between proteins of the ubiquinone complex (connected by black lines), encoded within introgressed area G among isolates from the second pandemic wave (shaded area). The numbers indicate the predicted confidence of functional interaction between two protein partners (ranging from 0-1) according to the STRING database. The images were modified from the STRING database (string-db.org).

Supplementary Tables

Table S1. List of isolates used to plot Figure S1 and for analysis of the ST-5 complex. (ND = not determined)

Isolate	Aliases/Accession Number	Country	Year	Strain Designation (Serogroup; PorA; FetA; ST; clonal complex)	ST (MLST)	Clonal complex (MLST)
A4/M1027	NIBSC:2803; Z1001; mlst082	USA	1937	A: P1.5-2,10: F1-5: ST-4 (cc4)	4	ST-4 complex
120M	NIBSC:2822; Z1035; mlst101	Pakistan	1967	A: P1.5-2,10: F5-1: ST-1 (cc1)	1	ST-1 complex
7891	NIBSC:2760; Z1054; mlst041	Finland	1975	A: P1.20,9: F3-1: ST-5 (cc5)	5	ST-5 complex
6748	NIBSC:2784; Z1073; mlst065	Canada	1971	A: P1.18-1,3: F5-1: ST-1 (cc1)	1	ST-1 complex
129E	NIBSC:2828; Z1092; mlst107	West Germany	1964	A: P1.5-2,10: F3-6: ST-1 (cc1)	1	ST-1 complex
139M	NIBSC:2795; Z1099; mlst074	Philippines	1968	A: P1.5-2,10: F5-1: ST-1 (cc1)	1	ST-1 complex
S3131	NIBSC:2813; Z1213; mlst092	Ghana	1973	A: P1.7,13-1: F1-5: ST-4 (cc4)	4	ST-4 complex
S4355	NIBSC:2806; Z1227; mlst085	Denmark	1974	A: P1.5-1,9: F3-1: ST-5 (cc5)	5	ST-5 complex
10	NIBSC:2825; Z1269; mlst104	Burkina Faso	1963	A: P1.7,13-1: F1-5: ST-4 (cc4)	4	ST-4 complex
20	NIBSC:2767; Z1275; mlst048	Niger	1963	A: P1.5-2,10: F1-7: ST-1 (cc1)	1	ST-1 complex
26	NIBSC:2764; Z1278; mlst045	Niger	1963	A: P1.7,13: F1-5: ST-4 (cc4)	4	ST-4 complex
255	NIBSC:2811; Z1318; mlst090	Burkina Faso	1966	A: P1.7-2,13-1: F1-5: ST-4 (cc4)	4	ST-4 complex
243	NIBSC:2779; Z1362; mlst060	Cameroon	1966	A: P1.7,13: F1-5: ST-4 (cc4)	4	ST-4 complex
393	NIBSC:2823; Z1392; mlst102	Greece	1968	A: P1.5-2,10: F5-1: ST-1 (cc1)	1	ST-1 complex
254	NIBSC:2812; Z5010; mlst091	Djibouti	1966	A: P1.5-2,10: F1-7: ST-1 (cc1)	1	ST-1 complex
S5611	NIBSC:2765; Z1466; mlst046	Australia	1977	A: P1.5-2,10: F5-1: ST-1 (cc1)	1	ST-1 complex
11-004	NIBSC:2826; Z1503; mlst105	China	1984	A: P1.20,9: F3-8: ST-5 (cc5)	5	ST-5 complex
IAL2229	NIBSC:2816; Z1506; mlst095	Brazil	1976	A: P1.20,9: F2-1: ST-5 (cc5)	5	ST-5 complex
F4698	NIBSC:2731; Z3515; mlst005	Saudi	1987	A: P1.20,9: F3-1: ST-5 (cc5)	5	ST-5 complex
F6124	NIBSC:2730; Z3524; mlst002	Chad	1988	A: P1.20,9: F3-1: ST-5 (cc5)	5	ST-5 complex
1014	NIBSC:2821; Z3667; mlst100	Sudan	1985	A: P1.7,13-1: F1-5: ST-4 (cc4)	4	ST-4 complex
H1964	NIBSC:2796; Z3771; mlst075	UK	1987	A: P1.20,9: F3-1: ST-5 (cc5)	5	ST-5 complex
NMBI	Z5463BC	France		A: P1.7,13-1: F1-5: ST-4 (cc4)	4	ST-4 complex
Z814	Z5463	Gambia		A: P1.7,13-1: F1-5: ST-4 (cc4)	4	ST-4 complex
153	NIBSC:2733; Z3905; mlst001	China	1966	A: P1.20,9: F3-1: ST-5 (cc5)	5	ST-5 complex
154	NIBSC:2766; Z3906; mlst047	China	1966	A: P1.20,9: F3-1: ST-6 (cc5)	6	ST-5 complex
80049	NIBSC:2793; Z4099; mlst072	China	1963	A: P1.5-2,10: F1-5: ST-5 (cc5)	5	ST-5 complex
243	Z5463PI	Gambia		A: P1.7,13-1: F1-5: ST-4 (cc4)	4	ST-4 complex
D8	NIBSC:2762; Z4186; mlst043	Mali	1990	A: P1.7,13-1: F1-5: ST-4 (cc4)	4	ST-4 complex
2059001	NIBSC:2745; Z4421; mlst026	Mali	1990	A: P1.7,13: F1-5: ST-4 (cc4)	4	ST-4 complex
BZ 133	NIBSC:2824; Z4665; mlst103	Netherlands	1977	B: P1.7,16: F5-1: ST-10300 (cc1)	10300	ST-1 complex
14/1455	NIBSC:2732; Z4717; mlst004	USSR	1970	A: P1.20,9: F3-1: ST-5 (cc5)	5	ST-5 complex
371	NIBSC:2769; Z4756; mlst050	India	1980	A: P1.5-2,10: F5-1: ST-1 (cc1)	1	ST-1 complex
690	NIBSC:2805; Z4757; mlst084	India	1980	A: P1.7,13-1: F1-5: ST-4 (cc4)	4	ST-4 complex
106	NIBSC:2808; Z5005; mlst087	Morocco	1967	A: P1.5-2,10: F5-1: ST-1 (cc1)	1	ST-1 complex
79128	NIBSC:2800; Z5035; mlst079	China	1979	A: P1.7-1,10: F5-5: ST-3 (cc1)	3	ST-1 complex

322/85	NIBSC:2810; Z5037; mlst089	East Germany	1985	A: P1.5-2,10: F5-2: ST-2 (cc1)	2	ST-1 complex
79126	NIBSC:2777; Z5043; mlst058	China	1979	A: P1.7-3,10-5: F5-5: ST-3 (cc1)	3	ST-1 complex
92001	NIBSC:2783; Z5826; mlst064	China	1992	A: P1.20,9: F3-1: ST-7 (cc5)	7	ST-5 complex
Z2491	C751; NIBSC:2763; Z6244; mlst044	Gambia	1983	A: P1.7,13-1: F1-5: ST-4 (cc4)	4	ST-4 complex
M13220		Philippines	2005	A: P1.20,9: F3-1: ST-7 (cc5)	7	ST-5 complex
M18575		Burkina Faso	2003	A: P1.ND,ND: F-ND: ST-2859 (cc5)	2859	ST-5 complex
WUE 2594		Germany	1991	A: P1.20,9: F1-21: ST-5 (cc5)	5	ST-5 complex
ERR051676				ND: P1.20,9: F3-1: ST-2859 (cc5)	2859	ST-5 complex
ERR051677				ND: P1.20,9: F3-1: ST-2859 (cc5)	2859	ST-5 complex
ERR051678				ND: P1.20,9: F3-1: ST-2859 (cc5)	2859	ST-5 complex
ERR051693				ND: P1.20,9: F3-1: ST-5 (cc5)	5	ST-5 complex
ERR051687				ND: P1.20,9: F3-1: ST-5 (cc5)	5	ST-5 complex
ERR052799				ND: P1.20,9: F3-1: ST-2859 (cc5)	2859	ST-5 complex
ERR052812				ND: P1.20,9: F3-1: ST-2859 (cc5)	2859	ST-5 complex
ERR052817				ND: P1.20,9: F3-1: ST-2859 (cc5)	2859	ST-5 complex
SRR513938				ND: P1.20,9: F3-1: ST-5 (cc5)	5	ST-5 complex
ERR052816				ND: P1.20,9: F3-1: ST-2859 (cc5)	2859	ST-5 complex
ERR052813				ND: P1.20,9: F3-1: ST-2859 (cc5)	2859	ST-5 complex
ERR052829				ND: P1.20,9: F3-1: ST-2859 (cc5)	2859	ST-5 complex
SRR513846				ND: P1.20,9: F3-1: ST-580 (cc5)	580	ST-5 complex
ERR052831				ND: P1.20,9: F3-1: ST-2859 (cc5)	2859	ST-5 complex
ERR052822				ND: P1.20,9: F3-1: ST-2859 (cc5)	2859	ST-5 complex
ERR052826				ND: P1.20,9: F3-1: ST-2859 (cc5)	2859	ST-5 complex
SRR513840				ND: P1.20,9: F3-1: ST-4789 (cc5)	4789	ST-5 complex
ERR052811				ND: P1.20,9: F3-1: ST-2859 (cc5)	2859	ST-5 complex
ERR052800				ND: P1.20,9: F3-1: ST-2859 (cc5)	2859	ST-5 complex
ERR052821				ND: P1.20,9: F3-1: ST-2859 (cc5)	2859	ST-5 complex
ERR052795				ND: P1.20,9: F3-1: ST-2859 (cc5)	2859	ST-5 complex
ERR052823				ND: P1.20,9: F3-1: ST-2859 (cc5)	2859	ST-5 complex
ERR052787				ND: P1.20,9: F3-1: ST-2859 (cc5)	2859	ST-5 complex
ERR052828				ND: P1.20,9: F3-1: ST-2859 (cc5)	2859	ST-5 complex
ERR052792				ND: P1.20,9: F3-1: ST-2859 (cc5)	2859	ST-5 complex
SRR514831				ND: P1.20,9: F3-1: ST-5 (cc5)	5	ST-5 complex
SRR513836				ND: P1.20,9: F3-1: ST-7 (cc5)	7	ST-5 complex
ERR052767				ND: P1.20,9: F3-1: ST-7 (cc5)	7	ST-5 complex
ERR052775				ND: P1.20,9: F3-1: ST-7 (cc5)	7	ST-5 complex
ERR052754				ND: P1.20,9: F3-1: ST-7 (cc5)	7	ST-5 complex
ERR052768				ND: P1.20,9: F3-1: ST-7 (cc5)	7	ST-5 complex
ERR052746				ND: P1.20,9: F3-1: ST-7 (cc5)	7	ST-5 complex
ERR052814				ND: P1.20,9: F3-1: ST-2859 (cc5)	2859	ST-5 complex
ERR052819				ND: P1.20,9: F3-1: ST-2859 (cc5)	2859	ST-5 complex
SRR513853				ND: P1.20,9: F3-1: ST-7 (cc5)	7	ST-5 complex
ERR052827				ND: P1.20,9: F3-1: ST-2859 (cc5)	2859	ST-5 complex
SRR513841				ND: P1.20,9: F-ND: ST-8428 (cc5)	8428	ST-5 complex
ERR052825				ND: P1.20,9: F3-1: ST-2859 (cc5)	2859	ST-5 complex
ERR052796				ND: P1.20,9: F3-1: ST-2859 (cc5)	2859	ST-5 complex

ERR052820	ND: P1.20,9: F3-1: ST-2859 (cc5)	2859	ST-5 complex
ERR052737	ND: P1.20,9: F3-1: ST-7 (cc5)	7	ST-5 complex
ERR052779	ND: P1.20,9: F3-1: ST-7 (cc5)	7	ST-5 complex
ERR052793	ND: P1.20,9: F3-1: ST-2859 (cc5)	2859	ST-5 complex
ERR052824	ND: P1.20,9: F3-1: ST-2859 (cc5)	2859	ST-5 complex
ERR052747	ND: P1.20,9: F3-1: ST-7 (cc5)	7	ST-5 complex
ERR052807	ND: P1.20,9: F3-1: ST-2859 (cc5)	2859	ST-5 complex
ERR052806	ND: P1.20,9: F3-1: ST-2859 (cc5)	2859	ST-5 complex
ERR052789	ND: P1.20,9: F3-1: ST-2859 (cc5)	2859	ST-5 complex
ERR052739	ND: P1.20,9: F3-1: ST-7 (cc5)	7	ST-5 complex
ERR052763	ND: P1.20,9: F3-1: ST-7 (cc5)	7	ST-5 complex
ERR052832	ND: P1.20,9: F3-1: ST-2859 (cc5)	2859	ST-5 complex
ERR052790	ND: P1.20,9: F3-1: ST-2859 (cc5)	2859	ST-5 complex
ERR052764	ND: P1.20,9: F3-1: ST-7 (cc5)	7	ST-5 complex
ERR052781	ND: P1.20,9: F3-1: ST-7 (cc5)	7	ST-5 complex
ERR052749	ND: P1.20,9: F3-1: ST-7 (cc5)	7	ST-5 complex
ERR052818	ND: P1.20,9: F3-1: ST-2859 (cc5)	2859	ST-5 complex
ERR052750	ND: P1.20,9: F3-1: ST-7 (cc5)	7	ST-5 complex
ERR052766	ND: P1.20,9: F3-1: ST-7 (cc5)	7	ST-5 complex
ERR052815	ND: P1.20,9: F3-1: ST-2859 (cc5)	2859	ST-5 complex
ERR052751	ND: P1.20,9: F3-1: ST-7 (cc5)	7	ST-5 complex
ERR052808	ND: P1.20,9: F3-1: ST-2859 (cc5)	2859	ST-5 complex
ERR052738	ND: P1.20,9: F3-1: ST-7 (cc5)	7	ST-5 complex
ERR052758	ND: P1.20,9: F3-1: ST-7 (cc5)	7	ST-5 complex
ERR052743	ND: P1.20,9: F3-1: ST-7 (cc5)	7	ST-5 complex
ERR052757	ND: P1.20,9: F3-1: ST-7 (cc5)	7	ST-5 complex
SRR513837	ND: P1.20,9: F3-1: ST-2859 (cc5)	2859	ST-5 complex
ERR052745	ND: P1.20,9: F3-1: ST-7 (cc5)	7	ST-5 complex
ERR052774	ND: P1.20,9: F3-1: ST-7 (cc5)	7	ST-5 complex
ERR052791	ND: P1.20,9: F3-1: ST-2859 (cc5)	2859	ST-5 complex
ERR052810	ND: P1.20,9: F3-1: ST-2859 (cc5)	2859	ST-5 complex
ERR052778	ND: P1.20,9: F3-1: ST-7 (cc5)	7	ST-5 complex
ERR052804	ND: P1.20,9: F3-1: ST-2859 (cc5)	2859	ST-5 complex
ERR052771	ND: P1.20,9: F3-1: ST-7 (cc5)	7	ST-5 complex
ERR052765	ND: P1.20,9: F3-1: ST-7 (cc5)	7	ST-5 complex
ERR052741	ND: P1.20,9: F3-1: ST-7 (cc5)	7	ST-5 complex
ERR052786	ND: P1.20,9: F3-1: ST-7 (cc5)	7	ST-5 complex
ERR052752	ND: P1.20,9: F3-1: ST-7 (cc5)	7	ST-5 complex
ERR052805	ND: P1.20,9: F3-1: ST-2859 (cc5)	2859	ST-5 complex
ERR052780	ND: P1.20,9: F3-1: ST-7 (cc5)	7	ST-5 complex
ERR052782	ND: P1.20,9: F3-1: ST-7 (cc5)	7	ST-5 complex
ERR052744	ND: P1.20,9: F3-1: ST-7 (cc5)	7	ST-5 complex
ERR052809	ND: P1.20,9: F3-1: ST-2859 (cc5)	2859	ST-5 complex
ERR052802	ND: P1.20,9: F3-1: ST-2859 (cc5)	2859	ST-5 complex
ERR052769	ND: P1.20,9: F3-1: ST-7 (cc5)	7	ST-5 complex
ERR052759	ND: P1.20,9: F3-1: ST-7 (cc5)	7	ST-5 complex

ERR052773	ND: P1.20,9: F3-1: ST-7 (cc5)	7	ST-5 complex
ERR052740	ND: P1.20,9: F3-1: ST-7 (cc5)	7	ST-5 complex
ERR052756	ND: P1.20,9: F3-1: ST-7 (cc5)	7	ST-5 complex
ERR052830	ND: P1.20,9: F3-1: ST-2859 (cc5)	2859	ST-5 complex
ERR052794	ND: P1.20,9: F3-1: ST-2859 (cc5)	2859	ST-5 complex
ERR052755	ND: P1.20,9: F3-1: ST-7 (cc5)	7	ST-5 complex
ERR052784	ND: P1.20,9: F3-1: ST-7 (cc5)	7	ST-5 complex
ERR052761	ND: P1.20,9: F3-1: ST-7 (cc5)	7	ST-5 complex
ERR052753	ND: P1.20,9: F3-1: ST-7 (cc5)	7	ST-5 complex
ERR052801	ND: P1.20,9: F3-1: ST-2859 (cc5)	2859	ST-5 complex
ERR052783	ND: P1.20,9: F3-1: ST-7 (cc5)	7	ST-5 complex
ERR052798	ND: P1.20,9: F3-1: ST-2859 (cc5)	2859	ST-5 complex
ERR052770	ND: P1.20,9: F3-1: ST-7 (cc5)	7	ST-5 complex
ERR052788	ND: P1.20,9: F3-1: ST-2859 (cc5)	2859	ST-5 complex
ERR052803	ND: P1.20,9: F3-1: ST-2859 (cc5)	2859	ST-5 complex
ERR052797	ND: P1.20,9: F3-1: ST-2859 (cc5)	2859	ST-5 complex
ERR052760	ND: P1.20,9: F3-1: ST-7 (cc5)	7	ST-5 complex
ERR052777	ND: P1.20,9: F3-1: ST-7 (cc5)	7	ST-5 complex
ERR052785	ND: P1.20,9: F3-1: ST-7 (cc5)	7	ST-5 complex
ERR052776	ND: P1.20,9: F3-1: ST-7 (cc5)	7	ST-5 complex
ERR052762	ND: P1.20,9: F3-1: ST-7 (cc5)	7	ST-5 complex
ERR052742	ND: P1.20,9: F3-1: ST-7 (cc5)	7	ST-5 complex
ERR052772	ND: P1.20,9: F3-1: ST-7 (cc5)	7	ST-5 complex
ERR052748	ND: P1.20,9: F3-1: ST-7 (cc5)	7	ST-5 complex
SRR606726	ND: P1.20,9: F3-1: ST-7 (cc5)	7	ST-5 complex
SRR606727	ND: P1.20,9: F3-1: ST-7 (cc5)	7	ST-5 complex
SRR606739	ND: P1.20,9: F3-1: ST-7 (cc5)	7	ST-5 complex
SRR606725	ND: P1.20,9: F3-1: ST-7 (cc5)	7	ST-5 complex
SRR606730	ND: P1.20,9: F3-1: ST-7 (cc5)	7	ST-5 complex
SRR606738	ND: P1.20,9: F3-1: ST-7 (cc5)	7	ST-5 complex
SRR606721	ND: P1.20,9: F3-1: ST-5 (cc5)	5	ST-5 complex
SRR606732	ND: P1.20,9: F3-1: ST-7 (cc5)	7	ST-5 complex
SRR606729	ND: P1.20,9: F3-1: ST-7 (cc5)	7	ST-5 complex
SRR606723	ND: P1.20,9: F3-1: ST-5 (cc5)	5	ST-5 complex
SRR606720	ND: P1.20,9: F3-1: ST-5 (cc5)	5	ST-5 complex
SRR606737	ND: P1.20,9: F3-1: ST-7 (cc5)	7	ST-5 complex
SRR606722	ND: P1.20,9: F3-1: ST-5 (cc5)	5	ST-5 complex
SRR606736	ND: P1.20,9: F3-1: ST-7 (cc5)	7	ST-5 complex
SRR606724	ND: P1.20,9: F3-1: ST-7 (cc5)	7	ST-5 complex
SRR606731	ND: P1.20,9: F3-1: ST-7 (cc5)	7	ST-5 complex
SRR606696	ND: P1.20,9: F3-1: ST-5 (cc5)	5	ST-5 complex
SRR606697	ND: P1.20,9: F2-1: ST-5 (cc5)	5	ST-5 complex
SRR606719	ND: P1.20,9: F3-1: ST-5 (cc5)	5	ST-5 complex
SRR606733	ND: P1.20-1,9: F3-1: ST-7 (cc5)	7	ST-5 complex
SRR606699	ND: P1.20,9: F3-1: ST-5 (cc5)	5	ST-5 complex
SRR606734	ND: P1.20-1,9: F3-1: ST-7 (cc5)	7	ST-5 complex

SRR606695			ND: P1.20,9: F3-1: ST-5 (cc5)	5	ST-5 complex
SRR606698			ND: P1.20,9: F2-1: ST-5 (cc5)	5	ST-5 complex
SRR606700			ND: P1.20,9: F3-1: ST-5 (cc5)	5	ST-5 complex
SRR606718			ND: P1.20,9: F3-1: ST-5 (cc5)	5	ST-5 complex
SRR606703			ND: P1.20,9: F3-1: ST-580 (cc5)	580	ST-5 complex
SRR606691			ND: P1.20,9: F3-1: ST-7 (cc5)	7	ST-5 complex
SRR606704			ND: P1.20,9: F3-1: ST-580 (cc5)	580	ST-5 complex
SRR606692			ND: P1.20,9: F3-1: ST-7 (cc5)	7	ST-5 complex
M11 240262	UK	2011	ND: P1.20,9: F3-1: ST-4789 (cc5)	4789	ST-5 complex

Table S2. Loci which contained alleles unique to the strains from the first pandemic wave.

Locus in reference genome	Product	Genome Position	Functional characterisation
NMA0408	putative integral membrane protein	377328	Metabolism
NMA0410	3-demethylubiquinone-9 3-methyltransferase	380455	Metabolism
NMA0411	putative homoserine kinase	381201	Metabolism
NMA0478	putative outer membrane peptidase	463713	Environmental Information Processing
NMA0537	putative integral membrane protein	517957	Unknown
NMA0606	hypothetical protein NMA0606	580081	Unknown
NMA0608	carbamoyl phosphate synthase small subunit	580546	Metabolism
NMA0621	putative riboflavin kinase/FMN adenylyltransferase	599508	Metabolism
NMA0708	putative hexosaminidase	696057	Metabolism
NMA0709	putative integral membrane protein	697199	Environmental Information Processing
NMA0710	putative periplasmic serine protease	698921	Metabolism
NMA0946	putative regulatory protein	914617	Environmental Information Processing
NMA1104	putative superoxide dismutase	1052308	Cellular processes
NMA1539	putative cation-transporting ATPase	1432649	Environmental Information Processing
NMA1571	iron/sulphur-binding oxidoreductase	1475160	Metabolism
NMA1673	putative integral membrane transporter	1595427	Environmental Information Processing
NMA1683	ClpB protein	1605805	Genetic Information Processing
NMA1708	hypothetical protein	1639365	Metabolism
NMA1748	glutaminyl-tRNA synthetase	1691533	Genetic Information Processing
NMA1997	hypothetical protein (pseudogene)	1936559	Other

Table S3. Loci which contained alleles unique to the strains from the second pandemic wave.

Locus in reference genome	Product	Genome Position	Functional characterisation
NMA0004	Fic/DOC family	2952	Other
NMA0005	NADH dehydrogenase I chain K	3561	Metabolism
NMA0006	NADH dehydrogenase I chain J	3863	Metabolism
NMA0093	Zinc transporter ZupT	93576	Environmental information processing
NMA0094	valyl-tRNA synthetase	94482	Genetic Information Processing
NMA0132	50S ribosome-binding GTPase	115873	Genetic Information Processing
NMA0185	capsule polysaccharide modification protein	167551	Metabolism
NMA0186	capsule polysaccharide modification protein	168947	Metabolism
NMA0241	electron transfer flavoprotein alpha-subunit	225154	Metabolism
NMA0258	UDP-N-acetylglucosamine 1-carboxyvinyltransferase	241392	Metabolism
NMA0260	putative integral membrane protein	244168	Unknown
NMA0280	Iron permease FTR1 family	265572	Environmental information processing
NMA0345	GTPase ObgE	320559	Genetic Information Processing
NMA0365	Permease	337526	Environmental information processing
NMA0442	Divalent cation transporter	415729	Environmental information processing
NMA0452	membrane transport solute-binding protein	426802	Environmental information processing
NMA0453	TonB dependent receptor (FetA)	428465	Antigen/virulence; Environmental Information Processing
NMA0478	outer membrane peptidase	463713	Other
NMA0480	aldehyde dehydrogenase A	468118	Metabolism
NMA0483	putative transcriptional regulator	470401	Genetic Information Processing
NMA0485	ABC transporter ATP binding protein	471431	Environmental information processing
NMA0486	ABC transport inner membrane subunit	472339	Environmental information processing
NMA0487	outer membrane transport protein	473166	Environmental information processing
NMA0488	periplasmic transport protein	473697	Environmental information processing
NMA0489	STAS domain	474320	Unknown
NMA0490	putative periplasmic/outer membrane protein	474619	Unknown
NMA0622	Isoleucine--tRNA ligase	600606	Genetic Information Processing
NMA0631	HNH endonuclease	610868	Other
NMA0658	Cytochrome C	644324	Metabolism
NMA0707	7-carboxy-7-deazaguanine synthase	694741	Metabolism
NMA0740	Unknown	733754	Unknown
NMA0741	ubiquinone biosynthesis protein UbiB	734253	Metabolism
NMA0745	putative periplasmic protein	737825	Unknown

NMA0746	thiamine biosynthesis protein	738066	Metabolism
NMA0747	Na(+)-translocating NADH-quinone reductase subunit F	739274	Metabolism
NMA0748	Na(+)-translocating NADH-quinone reductase subunit E	740505	Metabolism
NMA0749	Na(+)-translocating NADH-quinone reductase subunit D	741102	Metabolism
NMA0750	Na(+)-translocating NADH-quinone reductase subunit C	741728	Metabolism
NMA0751	Na(+)-translocating NADH-quinone reductase subunit B	742497	Metabolism
NMA0906	competence protein	881651	Other
NMA0925	Cytochrome C	897012	Metabolism
NMA1020	transcriptional regulator	981761	Genetic Information Processing
NMA1024	Adenylosuccinate synthetase	986609	Metabolism
NMA1084	Putative MetA-pathway of phenol degradation	1032148	Metabolism
NMA1120	Oxidoreductase	1070172	Other
NMA1263	Nudix family	1189567	Unknown
NMA1356	UDP-N-acetylmuramate:L-alanyl-gamma-D-glutamyl-meso-diaminopimelate ligase	1253355	Metabolism
NMA1416	riboflavin synthase alpha subunit	1314389	Metabolism
NMA1438	putative integral membrane protein	1331072	Unknown
NMA1572	pyridoxamine-5'-phosphate oxidase	1476300	Metabolism
NMA1573	pseudouridine synthase	1477432	Metabolism
NMA1574	integral membrane transporter	1478271	Environmental information processing
NMA1584	acetylornithine aminotransferase	1489480	Metabolism
NMA1589	putative type III restriction/modification system modification methylase (pseudogene)	1494377	Other
NMA1675	Transposase	1598453	Other
NMA1742	CTP synthase	1684464	Metabolism
NMA1748	glutaminyl-tRNA synthetase	1691533	Genetic Information Processing
NMA1771	histidinol-phosphate aminotransferase	1716562	Metabolism
NMA1838	DNA-binding protein	1781866	Unknown
NMA1891	Nitroreductase	1816250	Other
NMA1894	phosphoserine aminotransferase	1818798	Metabolism
NMA1896	N utilisation substance protein A	1820713	Genetic Information Processing
NMA1897	initiation factor IF2	1822242	Genetic Information Processing
NMA1923	Peptidase	1853690	Metabolism
NMA2052	aconitate hydratase	1996523	Metabolism
NMA2053	Sulfite exporter TauE/SafE	1999207	Environmental information processing
NMA2054	citrate synthase	2000156	Metabolism
NMA2055	Phosphoenolpyruvate phosphomutase	2001396	Metabolism
NMA2056	Permease	2002897	Environmental information processing

Table S4. Loci which contained alleles unique to the strains from the third pandemic wave.

Locus in reference genome	Product	Genome Position	Functional characterisation
NMA0035	Glutamate-ammonia-ligase adenylyltransferase	28626	Metabolism
NMA0049	RNA polymerase sigma factor	41790	Environmental Information Processing
NMA0066	dihydrodipicolinate reductase	60973	Metabolism
NMA0071	ribonuclease E	65414	Genetic Information Processing
NMA0080	ribosome recycling factor	79151	Genetic Information Processing
NMA0081	Isoprenyl transferase	79764	Metabolism
NMA0083	1-deoxy-D-xylulose 5-phosphate reductoisomerase	81366	Metabolism
NMA0135	elongation factor G	118817	Genetic Information Processing
NMA0174	unknown	157181	Unknown
NMA0280	Iron permease FTR1 family	265572	Environmental Information Processing
NMA0299	Transferrin binding protein-like solute binding protein	291661	Environmental Information Processing
NMA0350	Serine hydrolase	326157	Metabolism
NMA0408	Sulfatase	377328	Metabolism
NMA0477	para-aminobenzoate synthase component I	460532	Metabolism
NMA0519	ATP synthase beta chain	496617	Metabolism
NMA0521	glycyl-tRNA synthetase alpha chain	498802	Genetic Information Processing
NMA0522	unknown	499784	Unknown
NMA0634	unknown	618565	Unknown
NMA0650	pilus secretin	635588	Other
NMA0707	7-carboxy-7-deazaguanine synthase (CDG synthase)	694741	Metabolism
NMA0759	glycine cleavage system component H	750467	Metabolism
NMA0902	Ribosomal RNA small subunit methyltransferase A	871027	Genetic Information Processing
NMA0968	Phosphoribosylaminoimidazole-succinocarboxamide synthase	932217	Metabolism
NMA1000	ABC transporter	964511	Environmental Information Processing
NMA1001	phosphoglucomutase	965286	Metabolism
NMA1002	peptidyl-prolyl cis-trans isomerase B	966831	Genetic Information Processing
NMA1003	Transmembrane transporter	967459	Environmental Information Processing
NMA1003A	unknown	969207	Unknown
NMA1004	Peptidyl-tRNA hydrolase	969731	Genetic Information Processing
NMA1005	RnfH family Ubiquitin	970362	Genetic Information Processing
NMA1006	Polyketide cyclase / dehydrase and lipid transport	970633	Metabolism
NMA1007	ATP-dependent zinc metallopeptidase	971286	Genetic Information Processing

NMA1038	type I restriction-modification system protein	998259	Genetic Information Processing
NMA1107	putative membrane protein	1055468	Unknown
NMA1131	unknown	1079957	Unknown
NMA1134	putative lipoprotein	1082178	Unknown
NMA1136	homoserine O-acetyltransferase	1083718	Metabolism
NMA1137	50S ribosomal protein L36	1085479	Genetic Information Processing
NMA1138	additional 50S ribosomal protein L31	1085604	Genetic Information Processing
NMA1139	Methylenetetrahydrofolate reductase	1086078	Metabolism
NMA1140	5-methyltetrahydropteroyltriglutamate--homocysteine methyltransferase	1087095	Metabolism
NMA1141	Redoxin	1089650	Cellular Processes
NMA1142	dihydrolipoamide dehydrogenase	1090636	Metabolism
NMA1200	Putative surface fibril protein	1139032	Other
NMA1210	unknown	1153976	Unknown
NMA1249	ABC transporter	1175111	Environmental Information Processing
NMA1267	Gamma-glutamyl phosphate reductase	1195261	Metabolism
NMA1400	Methyltransferase small domain	1299257	Genetic Information Processing
NMA1445	GTPase HflX (GTP-binding protein HflX)	1339576	Genetic Information Processing
NMA1446	unknown	1340759	Unknown
NMA1447	unknown	1342192	Unknown
NMA1448	DNA repair protein RadC	1342990	Genetic Information Processing
NMA1449	Glutamate-cysteine ligase	1343790	Metabolism
NMA1451	putative lipoprotein	1346901	Unknown
NMA1452	3-isopropylmalate dehydratase small subunit	1347217	Metabolism
NMA1453	DNA modification methylase	1348033	Metabolism
NMA1465	tRNA 2-thiocytidine biosynthesis protein TtcA	1357496	Genetic Information Processing
NMA1472	protein-tyrosine-phosphatase	1365637	Environmental Information Processing
NMA1473	glycerate kinase	1366217	Metabolism
NMA1476	mercuric ion binding protein	1370357	Genetic Information Processing
NMA1478	polysaccharide modification protein	1371201	Metabolism
NMA1491	transcription-repair coupling factor	1387161	Genetic Information Processing
NMA1524	4-hydroxy-3-methylbut-2-en-1-yl diphosphate synthase	1419151	Metabolism
NMA1541	sel1 repeat	1437010	Unknown
NMA1542	tRNA (guanine-N(7)-)-methyltransferase	1438565	Genetic Information Processing
NMA1544	virus-related protein	1440251	Other
NMA1545	excinuclease ABC subunit B	1441083	Genetic Information Processing
NMA1547	peptidase	1445043	Metabolism
NMA1563	SUN-family protein	1466222	Genetic Information Processing
NMA1588	tRNA pseudouridine synthase B	1493137	Metabolism
NMA1591	type III restriction/modification system enzyme	1496471	Genetic Information Processing
NMA1592	L-lactate dehydrogenase	1499437	Metabolism
NMA1593	Transcriptional regulator	1500899	Genetic Information Processing
NMA1594	NifS-like aminotranfserase	1501374	Metabolism; Genetic Information

			Processing
NMA1620	cytolysin secretion ABC transporter	1523857	Environmental Information Processing
NMA1677	unknown	1600980	Unknown
NMA1683	ClpB protein	1605805	Genetic Information Processing
NMA1730	succinyl-diaminopimelate desuccinylase	1663067	Metabolism
NMA1748	glutaminyl-tRNA synthetase	1691533	Genetic Information Processing
NMA1766	acetolactate synthase isozyme III large subunit	1709994	Metabolism
NMA1850	Phage Mu protein F like protein	1788811	Other
NMA1882	transposase	1804208	Unknown
NMA1887	nitrite reductase, major outer membrane copper-containing protein	1810404	Metabolism
NMA1936	cytochrome	1868180	Metabolism
NMA1946	DNAase	1879996	Metabolism
NMA1947	L-asparaginase	1880890	Metabolism
NMA1948	DedA-family integral membrane protein	1881946	Unknown
NMA1949	Phosphoglucosamine mutase	1882819	Metabolism
NMA1964	glutamate dehydrogenase	1898767	Metabolism
NMA1975	integral membrane protein	1915001	Unknown
NMA2024	transferrin-binding protein A	1962649	Antigen/virulence
NMA2025	transferrin-binding protein B	1965468	Antigen/virulence
NMA2063	cell division protein	2011353	Genetic Information Processing
NMA2080	Chelatase	2028669	Unknown

Table S5. Loci which contained alleles unique to ST-2859 strains.

Locus in reference genome	Product	Genome Position	Functional characterisation
murA | NMAA_0011	UDP-N-acetylglucosamine 1-carboxyvinyltransferase	7582	Metabolism
glmS | NMAA_0021	glucosamine--fructose-6-phosphate aminotransferase [isomerizing]	21789	Metabolism
thiB | NMAA_0029	Thiamine transport system substrate-binding protein	32717	Environmental information processing
NMAA_0030	Mechanosensitive ion channel	33812	Environmental information processing
NMAA_0031	Competence-damaged protein (CinA family)	34686	Unknown
msrAB | NMAA_0032	peptide methionine sulfoxide reductase (putative pilin biogenesis)	35273	Cellular processes
ftsY | NMAA_0033	probable signal recognition particle protein	36986	Environmental information processing
NMAA_0034	GIY-YIG catalytic domain	38840	Unknown
NMAA_0100	putative transcriptional activator protein METR	113047	Genetic information processing
fetA | NMAA_0164	TonB dependent receptor (FetA)	193640	Antigenic
NMAA_0299	ATP-binding protein involved in chromosome partitioning	353893	Cellular processes
NMAA_0300	putative membrane-associated thioredoxin	355325	Cellular processes
pglD | NMAA_0321	pilin glycosylation protein pglD	387460	Antigenic
pglC | NMAA_0322	pilin glycosylation protein pglC	389418	Antigenic
pglB1 | NMAA_0323	pilin glycosylation protein pglB1	390723	Antigenic
NMAA_0337	GTP binding protein engB	409695	Cellular processes
cyc | NMAA_0338	Cytochrome C	410530	Metabolism
NMAA_0339	Cytochrome C biogenesis protein	411367	Metabolism
ccsA | NMAA_0340	Cytochrome C biogenesis protein	413375	Metabolism
NMAA_0341	tRNA N6-adenosine threonylcarbamoyltransferase (EC 2.3.1.-)	414679	Metabolism
rluF | NMAA_0635	ribosomal large subunit pseudouridine synthase F	749433	Metabolism
NMAA_0636	ATP-NAD kinase	750216	Metabolism
NMAA_0637	None	751126	Unknown
NMAA_0638	NADH(P)-binding	751743	Unknown
murB | NMAA_0640	UDP-N-acetylenolpyruvoylglucosamine reductase (EC 1.3.1.98)	753236	Metabolism
NMAA_0641	multidrug efflux protein	754454	Environmental information processing
hisZ | NMAA_0642	ATP phosphoribosyltransferase regulatory subunit	756168	Metabolism
purA | NMAA_0643	Adenylosuccinate synthetase	757422	Metabolism
NMAA_0644	None	758839	Unknown
NMAA_0645	None	759132	Unknown
NMAA_0646	None	759583	Unknown
NMAA_0647	None	760110	Unknown

NMAA_0648	None	760563	Unknown
NMAA_0649	None	760708	Unknown
adk | NMAA_0651	adenylate kinase	762625	Metabolism
rfaE1 | NMAA_0653	pfkB family carbohydrate kinase (BIGS: D-beta-D-heptose-7-phosphate kinase)	764593	Metabolism
NMAA_0654	Cytosine-specific methyltransferase	765601	Metabolism
dnaB | NMAA_0710	DnaB-like helicase C terminal domain	823174	Genetic information processing
NMAA_0805	putative phage repressor	930949	Other
NMAA_0886	Putative phage tail fiber protein	1007824	Other
NMAA_0888	None	1010855	Unknown
NMAA_0889	Caudovirales tail fibre assembly protein	1011222	Other
NMAA_0890	None	1011806	Unknown
NMAA_0891	None	1012099	Unknown
NMAA_0892	Putative bacterial lipoprotein (DUF799)	1013947	Unknown
NMAA_0893	None	1014591	Unknown
NMAA_0894	Curli production assembly/transport component CsgG	1014969	Environmental information processing
NMAA_0895	short chain dehydrogenase	1015805	Metabolism
NMAA_0896	Flavin containing amine oxidoreductase	1016929	Metabolism
NMAA_0996	putative type III restriction-modification system enzyme Res	1134598	Cellular processes
NMAA_1049	Sulfite exporter TauE/SafE	1204715	Environmental information processing
NMAA_1067	Fumarylacetoacetate (FAA) hydrolase family	1225027	Metabolism
gdhB | NMAA_1182	Glutamate dehydrogenase	1387610	Metabolism
ackA1 | NMAA_1212	acetate kinase	1424286	Metabolism
lbpB | NMAA_1234	lactoferrin-binding protein	1454533	Antigenic
pyrG | NMAA_1235	CTP synthase	1459282	Metabolism
fadD1 | NMAA_1236	long-chain-fatty-acid--CoA-ligase	1461028	Metabolism
trmU | NMAA_1237	tRNA (5-methylaminomethyl-2-thiouridylate)-methyltransferase	1462769	Genetic information processing
dgk | NMAA_1239	diacylglycerol kinase	1464586	Metabolism
acnB | NMAA_1253	aconitate hydratase	1478878	Metabolism
NMAA_1298	None	1538271	Unknown
NMAA_1383	None	1621406	Unknown
NMAA_1495	Nitrilase	1752253	Metabolism
murF | NMAA_1519	UDP-MurNAc-pentapeptide synthetase	1780672	Metabolism
mafA2 | NMAA_1550	adhesin MafA2	1828085	Antigenic
mafB2 | NMAA_1551	adhesin MafB2	1829030	Antigenic
NMAA_1553	None	1830920	Unknown
NMAA_1554	None	1831592	Unknown
NMAA_1555	None	1831846	Unknown
NMAA_1556	Suppressor of fused protein (SUFU)	1831985	Environmental information processing
NMAA_1557	None	1832666	Unknown
NMAA_1558	None	1833347	Unknown
NMAA_1559	Regulator of ribonuclease activity B	1833690	Genetic information

			processing; metabolism
NMAA_1560	None	1834072	Unknown
NMAA_1561	Suppressor of fused protein (SUFU)	1834426	Environmental information processing
NMAA_1562	None	1835104	Unknown
NMAA_1563	None	1835834	Unknown
NMAA_1564	None	1836359	Unknown
NMAA_1566	None	1837213	Unknown
frpD | NMAA_1567	RTX iron-regulated protein FrpC	1838725	Antigenic
NMAA_1568	GIY-YIG catalytic domain	1839879	Unknown
NMAA_1707	None	1976980	Unknown
nuoB | NMAA_1739	NADH dehydrogenase I chain B	2006433	Metabolism
gidB | NMAA_1788	Ribosomal RNA small subunit methyltransferase G	2069801	Genetic information processing
NMAA_1789	Putative integral membrane protein	2070582	Unknown
NMAA_1790	Fusaric acid resistance protein family	2071324	Other
NMAA_1914	Alanine racemase, N-terminal domain	2195758	Metabolism

Table S6. Loci of genes missing in ST-2859 and/or third pandemic wave isolates but present in the ancestral strains.

	Genome position	Locus in reference genome	Product	Functional characterisation
**Genes missing in ST-2859 isolates**				
A	38840	NMAA_0034	GIY-YIG catalytic domain	Unknown
E	759132	NMAA_0645	None characterised	Unknown
E	759583	NMAA_0646	None characterised	Unknown
E	760110	NMAA_0647	None characterised	Unknown
E	760563	NMAA_0648	None characterised	Unknown
E	760708	NMAA_0649	None characterised	Unknown
G	1454533	lbpB | NMAA_1234	Lactoferrin-binding protein	Antigenic
H	1831592	NMAA_1554	None characterised	Unknown
H	1831846	NMAA_1555	None characterised	Unknown
H	1831985	NMAA_1556	Suppressor of fused protein	Unknown
H	1833347	NMAA_1558	None characterised	Unknown
H	1833690	NMAA_1559	None characterised	Unknown
H	1834072	NMAA_1560	None characterised	Unknown
H	1834426	NMAA_1561	Suppressor of fused protein	Unknown
**Genes missing in third pandemic wave**				
G	1440251	NMA1544	Virus-related protein	Other

Table S7. Alleles of major outer membrane antigens of 153 genomes of the ST-5 complex.

Isolate	PorA	PorB	FetA	FHBp	NadA	opcA	TbpA	TbpB	NMA1332	GNA33	GNA1220
NIBSC:2760; Z1054; mlst041	P1.20,9	3-27	F3-1	5	7	3	9	9	3	7	45
NIBSC:2806; Z1227; mlst085	P1.5-1,9	3-27	F3-1	5	7	3	9	14	3	7	45
NIBSC:2826; Z1503; mlst105	P1.20,9	3-61	F3-1	5	7	3	9	106	3	17	45
NIBSC:2816; Z1506; mlst095	P1.20,9	3-27	F3-1	5	7	3	9	14	3	7	45
NIBSC:2731; Z3515; mlst005	P1.20,9	3-47	F3-1	5	16	3	9	14	3	19	45
NIBSC:2730; Z3524; mlst002	P1.20,9	3-47	F3-1	5	7		9		3	19	45
NIBSC:2796; Z3771; mlst075	P1.20,9	3-47	F3-1	5	7	3	9	19	3	19	45
NIBSC:2733; Z3905; mlst001	P1.20,9	3-47	F3-1	5	7	3	19	24	3	19	45
NIBSC:2766; Z3906; mlst047	P1.20,9	3-47	F3-1		7	3	9	14	3	19	45
NIBSC:2793; Z4099; mlst072	P1.5-2,10	3-56	F1-5	5	7	3	89	121	3	23	48
NIBSC:2732; Z4717; mlst004	P1.20,9	3-47	F3-1	5	7	3	9	82	3	19	45
NIBSC:2783; Z5826; mlst064	P1.20,9	3-62	F3-1	39	7	3	62	85	3	19	45
FR774048	P1.20,9	3-47	F1-21	5		3	9	14	3	19	2
ERR051676	P1.20,9	3-47	F3-1	39	7	3	1453		1		45
ERR051677	P1.20,9	3-47	F3-1	39	7	3	1453		1	19	45
ERR051678	P1.20,9	3-47	F3-1	39	7	3	1453		1		45
ERR051693	P1.20,9	3-47	F3-1		7	3	9	14	3	19	45
ERR051687	P1.20,9	3-47	F3-1	5	7	3	9		3		45
ERR052799	P1.20,9	3-47	F3-1	39	7	3	1453	85	1	19	45
ERR052812	P1.20,9	3-47	F3-1	39	7	3	1453	85	1	19	45
ERR052817	P1.20,9	3-47	F3-1	39	7	3	1453	85	1	19	45
SRR513938	P1.20,9	3-47	F3-1	5	7	3	9	14	3	19	45
ERR052816	P1.20,9	3-47	F3-1	39	7	3		85	1	19	45
ERR052813	P1.20,9	3-47	F3-1	39	7	3	1453	85	1	19	45
ERR052829	P1.20,9	3-47	F3-1	39	7	3	1453	85	1	19	45
SRR513846	P1.20,9	3-47	F3-1	5	7	3	9	14	3	19	45
ERR052831	P1.20,9	3-47	F3-1	39	7	3	1453	85	1	19	45
ERR052822	P1.20,9	3-47	F3-1	39	7	3	1453	85	1	19	45
ERR052826	P1.20,9	3-47	F3-1	39	7	3	1453	85	1	19	45
SRR513840	P1.20,9	3-142	F3-1	39	7	3	1453	85	3	19	45
ERR052811	P1.20,9	3-47	F3-1	39	7	3	1453	85	1	19	45
ERR052800	P1.20,9	3-47	F3-1	39	7	3	1453	85	1	19	45
ERR052821	P1.20,9	3-47	F3-1	39	7	3	1453	85	1	19	45
ERR052795	P1.20,9	3-47	F3-1	39	7	3	1453	85	1	19	45
ERR052823	P1.20,9	3-47	F3-1	39	7	3	1453	85	1	19	45
ERR052787	P1.20,9	3-47	F3-1	39	7	3	1453	85	1	19	45
ERR052828	P1.20,9	3-47	F3-1	39	7	3	1453	85	1	19	45

ERR052792	P1.20,9	3-47	F3-1	39	7	3	1453	85	1	19	45
SRR514831	P1.20,9	3-47	F3-1	5	7	3	9	14	3	19	45
SRR513836	P1.20,9	3-47	F3-1	39	7	3	1453	85	3	19	45
ERR052767	P1.20,9	3-47	F3-1	39	7	3	1453	85	3	19	45
ERR052775	P1.20,9	3-47	F3-1	39	7	3	1453	85	3	19	45
ERR052754	P1.20,9	3-47	F3-1	39	7	3	1453	85	3	19	45
ERR052768	P1.20,9	3-47	F3-1	39	7	3	1453	85	3	19	45
ERR052746	P1.20,9	3-47	F3-1	39	7	3	1453	85	3	19	45
ERR052814	P1.20,9	3-47	F3-1	39	7	3	1453	85	1	19	45
ERR052819	P1.20,9	3-47	F3-1	39	7	3	1453	85	1	19	45
SRR513853	P1.20,9	3-47	F3-1	39	7	3	1453	85	3	19	45
ERR052827	P1.20,9	3-47	F3-1	39	7	3	1453	85	1	19	45
SRR513841	P1.20,9	3-142	F-ND	39	7	3	1453		3	19	45
ERR052825	P1.20,9	3-47	F3-1	39	7	3	1453	85	1	19	45
ERR052796	P1.20,9	3-47	F3-1	39	7	3	1453	85	1	19	45
ERR052820	P1.20,9	3-47	F3-1	39	7	3	1453	85	1	19	45
ERR052737	P1.20,9	3-47	F3-1	39	7	3	1453	85	3	19	45
ERR052779	P1.20,9	3-47	F3-1	39	7	3	1453	85	3	19	45
ERR052793	P1.20,9	3-47	F3-1	39	7	3	1453	85	1	19	45
ERR052824	P1.20,9	3-47	F3-1	39	7	3	1453	85	1	19	45
ERR052747	P1.20,9	3-47	F3-1	39	7	3	1453	85	3	19	45
ERR052807	P1.20,9	3-47	F3-1	39	7	3	1453	85	1	19	45
ERR052806	P1.20,9	3-47	F3-1	39	7	3	1453	85	1	19	45
ERR052789	P1.20,9	3-47	F3-1	39	7	3	1453	85	1	19	45
ERR052739	P1.20,9	3-47	F3-1	39	7	3	1453	85	3	19	45
ERR052763	P1.20,9	3-47	F3-1	39	7	3	1453	85	3	19	45
ERR052832	P1.20,9	3-47	F3-1	39	7	3	1453	85	1	19	45
ERR052790	P1.20,9	3-47	F3-1	39	7	3	1453	85	1	19	45
ERR052764	P1.20,9	3-47	F3-1	39	7	3	1453	85	3	19	45
ERR052781	P1.20,9	3-47	F3-1	39	7	3	1453	85	3	19	45
ERR052749	P1.20,9	3-47	F3-1	39	7	3	1453	85	3	19	45
ERR052818	P1.20,9	3-47	F3-1	39	7	3	1453	85	1	19	45
ERR052750	P1.20,9	3-47	F3-1	39	7	3	1453	85	3	19	45
ERR052766	P1.20,9	3-47	F3-1	39	7	3	1453	85	3	19	45
ERR052815	P1.20,9	3-47	F3-1	39	7	3	1453	85	1	19	45
ERR052751	P1.20,9	3-47	F3-1	39	7	3	1453	85	3	19	45
ERR052808	P1.20,9	3-47	F3-1	39	7	3	1453	85	1	19	45
ERR052738	P1.20,9	3-47	F3-1	39	7	3	1453	85	3	19	45
ERR052758	P1.20,9	3-47	F3-1	39	7	3	1453	85	3	19	45
ERR052743	P1.20,9	3-47	F3-1	39	7	3	1453	85	3	19	45
ERR052757	P1.20,9	3-47	F3-1	39	7	3	1453	85	3	19	45
SRR513837	P1.20,9	3-47	F3-1	39	7	3	1453	85	1	19	45
ERR052745	P1.20,9	3-47	F3-1	39	7	3	1453	85	3	19	45
ERR052774	P1.20,9	3-47	F3-1	39	7	3	1453	85	3	19	45
ERR052791	P1.20,9	3-47	F3-1	39	7	3	1453	85	1	19	45
ERR052810	P1.20,9	3-47	F3-1	39	7	3	1453	85	1	19	45

ERR052778	P1.20,9	3-47	F3-1	39	7	3	1453	85	3	19	45
ERR052804	P1.20,9	3-47	F3-1	39	7	3	1453		1	19	45
ERR052771	P1.20,9	3-47	F3-1	39	7	3	1453	85	3	19	45
ERR052765	P1.20,9	3-47	F3-1	39	7	3	1453	85	3	19	45
ERR052741	P1.20,9	3-47	F3-1	39	7	3	1453	85	3	19	45
ERR052786	P1.20,9	3-47	F3-1	39	7	3	1453	85	3	19	45
ERR052752	P1.20,9	3-47	F3-1	39	7	3	1453	85	3	19	45
ERR052805	P1.20,9	3-47	F3-1	39	7	3	1453	85	1	19	45
ERR052780	P1.20,9	3-47	F3-1	39	7	3	1453	85	3	19	45
ERR052782	P1.20,9	3-47	F3-1	39	7	3	1453	85	3	19	45
ERR052744	P1.20,9	3-47	F3-1	39	7	3	1453	85	3	19	45
ERR052809	P1.20,9	3-47	F3-1	39	7	3	1453		1	19	45
ERR052802	P1.20,9	3-47	F3-1	39	7	3	1453	85	1	19	45
ERR052769	P1.20,9	3-47	F3-1	39	7	3	1453	85	3	19	45
ERR052759	P1.20,9	3-47	F3-1	39	7	3	1453		3	19	45
ERR052773	P1.20,9	3-47	F3-1	39	7	3	1453	85	3	19	45
ERR052740	P1.20,9	3-47	F3-1	39	7	3	1453	85	3	19	45
ERR052756	P1.20,9	3-47	F3-1	39	7	3	1453	85	3	19	45
ERR052830	P1.20,9	3-47	F3-1	39	7	3	1453	85	1	19	45
ERR052794	P1.20,9	3-47	F3-1	39	7	3	1453	85	1	19	45
ERR052755	P1.20,9	3-47	F3-1	39	7	3	1453	85	3	19	45
ERR052784	P1.20,9	3-47	F3-1	39	7	3	1453	85	3	19	45
ERR052761	P1.20,9	3-47	F3-1	39	7	3	1453	85	3	19	45
ERR052753	P1.20,9	3-47	F3-1	39	7	3	1453	85	3	19	45
ERR052801	P1.20,9	3-47	F3-1	39	7	3	1453	85	1	19	45
ERR052783	P1.20,9	3-47	F3-1	39	7		1453	85	3	19	45
ERR052798	P1.20,9	3-47	F3-1	39	7	3	1453	85	1	19	45
ERR052770	P1.20,9	3-47	F3-1	39	7	3	1453	85	3	19	45
ERR052788	P1.20,9	3-47	F3-1	39	7	3	1453	85	1	19	45
ERR052803	P1.20,9	3-47	F3-1	39	7	3	1453	85	1	19	45
ERR052797	P1.20,9	3-47	F3-1	39	7		1453	85	1	19	45
ERR052760	P1.20,9	3-47	F3-1	39	7		1453	85	3	19	45
ERR052777	P1.20,9	3-47	F3-1	39	7		1453	85	3	19	45
ERR052785	P1.20,9	3-47	F3-1	39	7		1453	85	3	19	45
ERR052776	P1.20,9	3-47	F3-1	39	7	3	1453	85	3	19	45
ERR052762	P1.20,9	3-47	F3-1	39	7	3	1453	85	3	19	45
ERR052742	P1.20,9	3-47	F3-1	39	7	3	1453	85	3	19	45
ERR052772	P1.20,9	3-47	F3-1	39	7	3	1453	85	3	19	45
ERR052748	P1.20,9	3-47	F3-1	39	7	3	1453	85	3	19	45
SRR606726	P1.20,9	3-47	F3-1	39	7	3	1453	85	3		45
SRR606727	P1.20,9	3-47	F3-1	39	7	3	1453	85	3		45
SRR606739	P1.20,9	3-47	F3-1	39	7	3	1453	85	3	19	45
SRR606725	P1.20,9	3-47	F3-1	39	7	3	1453	85	3	19	45
SRR606730	P1.20,9	3-47	F3-1	39	7	3	1453	85	3	19	45
SRR606738	P1.20,9	3-47	F3-1	39	7	3	1453	85	3	19	45
SRR606721	P1.20,9	3-47	F3-1	5	7	3			3	19	45

SRR606732	P1.20,9	3-47	F3-1	39	7	3	1453	85	3	19	45
SRR606729	P1.20,9	3-47	F3-1	39	7	3	1453	85	3	19	45
SRR606723	P1.20,9	3-47	F3-1	5	7	3	9	14	3	19	45
SRR606720	P1.20,9	3-47	F3-1	5	7	3			3	19	45
SRR606737	P1.20,9	3-47	F3-1	39	7	3	1453	85	3	19	45
SRR606722	P1.20,9	3-47	F3-1	5	7	3	9	14	3	19	45
SRR606736	P1.20,9	3-47	F3-1	39	7	3	1453	85	3	19	45
SRR606724	P1.20,9	3-47	F3-1	39	7	3	1453	85	3	19	45
SRR606731	P1.20,9	3-47	F3-1	39	7	3	1453	85	3	19	45
SRR606696	P1.20,9	3-47	F3-1	5	7	3	9	14	3	19	45
SRR606697	P1.20,9	3-27	F2-1	5	7	3	9		3	7	45
SRR606719	P1.20,9	3-47	F3-1	5	7	3			3	19	45
SRR606733	P1.20-1,9	3-47	F3-1	39	7	3	1453	85	3	19	45
SRR606699	P1.20,9	3-27	F3-1	5	7	3	9	14	3	7	45
SRR606734	P1.20-1,9	3-47	F3-1	39	7	3	1453	85	3	19	45
SRR606695	P1.20,9	3-47	F3-1		7	3	9	14	3	19	45
SRR606698	P1.20,9	3-27	F3-1	5	7	3	9		3	7	45
SRR606700	P1.20,9	3-27	F3-1	5	7	3	9	14	3	7	45
SRR606718	P1.20,9	3-47	F3-1	5	7	3			3	19	45
SRR606703	P1.20,9	3-47	F3-1	5	7	3	9	14	3	19	45
SRR606691	P1.20,9	3-47	F3-1	39	7	3	1453	85	3	19	45
SRR606704	P1.20,9	3-47	F3-1	5	7	3	9	14	3	19	45
SRR606692	P1.20,9	3-47	F3-1	39	7	3	1453	85	3	19	45
M11 240262	P1.20,9	3-47	F3-1	39	7	3	587	654	3	19	45

Supplementary Text

When all 153 genomes were analysed against reference strain Z2491, identical alleles across all isolates were found at 352 loci including the reference genome, and 103 loci excluding the reference genome loci (out of 1993 annotated loci present in the reference genome). Variable alleles were found at 999 loci, truncated sequences at one or more isolates were found at 528 loci, and 56 loci were paralogous and excluded from further analysis.

When isolates from the third pandemic wave and ST-2859 were analysed against reference strain WUE 2594, identical alleles across all isolates were found at 948 loci including the reference genome, and 109 loci excluding the reference genome loci (out of 2070 annotated loci present in the reference genome). Variable alleles were found at 560 loci, truncated sequences at one or more isolates were found at 392 loci, and 37 loci were paralogous and excluded from further analysis.
